# Kyoh^®^ Rocket Leaf Extract Regulates Proliferation and VEGF and FGF7 Expression in Human Dermal Follicle Papilla Cells

**DOI:** 10.3390/molecules30071489

**Published:** 2025-03-27

**Authors:** Adal Mena-García, Justyna M. Meissner, David Pajuelo, María Inés Morán-Valero, Ana Cristos, Marina Díez-Municio, Jose Luis Mullor

**Affiliations:** 1 Pharmactive Biotech Products S.L.U., Faraday 7, 28049 Madrid, Spain; ines.moran@pharmactive.eu (M.I.M.-V.); acristos@pharmactive.eu (A.C.); mdiez@pharmactive.eu (M.D.-M.); 2 Bionos S.L., Biopolo La Fe, Hospital La Fe, 46026 Valencia, Spain; jmeissner@bionos.es (J.M.M.); davidpajuelogamez@gmail.com (D.P.); jlmullor@bionos.es (J.L.M.)

**Keywords:** alopecia, rocket, hydroalcoholic extract, flavonols, dermal follicle papilla cells, VEGF, FGF7

## Abstract

Androgenetic alopecia is the most common cause of hair loss for women and men. Current treatments for androgenetic alopecia, such as those based on drugs like Minoxidil, Finasteride, or Dutasteride, have been associated with a variety of side effects, such as irritation, contact dermatitis, scalp pruritus, burning, etc. In this regard, plant extracts have emerged as promising alternatives to available chemical-based treatments for androgenetic alopecia given their efficacy, customer acceptability, and potentially minimized side effects. In this study, we evaluated the efficacy of Kyoh^®^, an extract from rocket leaves, as a treatment to improve the signs of androgenetic alopecia. We found that Kyoh^®^ contained 2.1% total flavonoids, with kaempferol, quercetin, and isorhamnetin diglucosides being the most abundant. Additionally, Kyoh^®^ showed a stimulating effect on the growth of human dermal follicle papilla cells in laboratory conditions. Most importantly, Kyoh^®^ enhanced the gene expression of the hair growth-associated growth factors VEGF (Vascular Endothelial Growth Factor) and FGF7 (Fibroblast Growth Factor 7). Specifically, VEGF expression increased by 60.7% after 4 h and 267.3% after 24 h, while FGF7 expression increased by 50.3% after 4 h and 244.3% after 24 h, indicating both a rapid induction of gene expression and a sustained effect lasting at least one day. Moreover, Kyoh^®^ increased the gene expression of NRF2 (Nuclear factor erythroid 2-related factor 2) by 71.2%, which encodes for a protein participating in the antioxidant response. Overall, our study shows that flavonol-rich rocket extract (Kyoh^®^) is a promising treatment for promoting hair growth, demonstrated by its proliferation-promoting effect, potential antioxidant priming, and induction of the expression of growth factors associated with hair growth and health.

## 1. Introduction

Androgenetic alopecia, also known as male or female pattern baldness, is the most common type of hair loss, affecting women (50% of menopausal women and 25% of women of childbearing age) and men (over 70%) [1]. This condition is characterized by a progressive thinning of scalp hair, which follows a specific pattern in both men and women. Androgenetic alopecia seems to occur due to a susceptibility of hair follicles to shrinkage as a result of a combination of different factors.

The pathogenesis of androgenetic alopecia is primarily driven by genetic predisposition, with numerous studies indicating that individuals with a family history of the condition are at a higher risk of developing it [2]. The inheritance pattern is complex and polygenic, meaning multiple genes contribute to the susceptibility of hair follicle sensitivity to hormones. On the other side, hormonal factors, particularly the androgen hormone dihydrotestosterone (DHT), play a critical role in the development of androgenetic alopecia [3]. DHT is derived from testosterone through the action of the enzyme 5-alpha-reductase. Hair follicles in individuals predisposed to androgenetic alopecia are more sensitive to DHT, which binds to androgen receptors in the hair follicles and shortens the anagen (growth) phase of the hair cycle, resulting in follicular atrophy and hair loss [4]. The interplay between these genetic and hormonal factors shapes the hair cycle and results in the characteristic patterns of hair thinning and loss associated with androgenetic alopecia [2,4,5,6].

Androgenetic alopecia is a chronic condition that is quite challenging to treat. Currently, there are only two US Food and Drug Administration (FDA)-approved drugs for the condition: topical minoxidil and oral finasteride. However, numerous non-FDA-approved treatments have been shown to be effective in treating androgenetic alopecia in various clinical studies. The therapies that showed efficacy in the treatment of androgenetic alopecia are based on different molecular targets and processes, such as arteriolar vasodilatation and induction of cell proliferation through VEGF expression (Minoxidil [7]), inhibition of 5-alpha-reductases to block formation of DHT (Finasteride and Dutasteride [8,9,10], prolongation of anagen phase via a prostaglandin analog (Latanoprost [11]) or antiandrogenic activity to decrease/block testosterone (spironolactone, flutamide, bicalutamide [12]). However, these treatments are associated with a variety of side effects such as headaches, hypertrichosis, irritation, contact dermatitis, scalp pruritus, burning, and erythema, sexual dysfunction, altered libido, gynecomastia, mood changes, dizziness, hypertrichosis, etc. [12].

Given the inconsistent results and dissatisfactory adverse effects associated with the current treatments, natural products can provide an alternative option for hair care as treatment doses are lower, which reduces side effects, and all active molecules are of natural origin and thus act on signaling pathways in a “natural” way, which also helps reduce side effects. Additionally, natural products offer ease and comfort to people who do not prefer chemically manufactured oral pills or topical agents. In this regard, plant extracts have emerged as promising treatments for androgenetic alopecia due to their natural bioactive compounds that can potentially promote hair growth and inhibit hair loss, offering a natural alternative or complementary approach to traditional treatments for androgenetic alopecia, potentially minimizing side effects [13]. Numerous clinical studies have demonstrated that treatments based on plant extracts might lead to the improvement of androgenetic alopecia by increasing total hair count [14,15,16,17,18,19,20], reducing terminal hair growth [21], increasing hair diameter [17,18,19,22,23], increasing growth rate [23], reduced hair loss [24], promote hair growth, and/or inhibit hair loss. These treatments differ in the nature of the plant extract and the mechanism of action [22].

The plant derivatives of the genus Eruca (such as rocket or Eruca sativa) have been shown to have antioxidant properties, potential anticarcinogenic activity, and other health benefits [25,26]. In particular, rocket oil has been used as a hair tonic to prevent hair loss [27] and proven to increase the hair length and weight of the newly grown hair [28]. Moreover, rocket leaves are rich in bioactive compounds like flavonols and glucosinolates, which have shown hair growth-promoting activities in in vitro studies when present in other natural sources [26,29,30,31]. Therefore, in this study, we hypothesize that Kyoh^®^, an extract from rocket leaves, may improve the signs of androgenetic alopecia at a molecular level. Consequently, we characterized the bioactive composition of the sample and analyzed how the extract acted on a molecular level regulating gene expression of androgenetic alopecia-related genes.

## 2. Results

### 2.1. Characterization of Kyoh^®^ Extract by HPLC-DAD-MS

Considering the chromatographic retention, the high-resolution mass spectrometry data, and the UV spectra (Figure S1) obtained by HPLC-DAD-TOF, we were able to characterize the bioactive compounds profile of Kyoh^®^ extract (Table 1). This profile consisted of the presence of several flavonoids, all of which are from the flavonol group, and some glucosinolates. The flavonols found in the commercial extract principally consisted of mono- and di-glucosides of kaempferol, quercetin, and isorhamnetin. Moreover, other flavonol glycosides, such as rutin, kaempferol-3-O-sophoroside, and two quercetin trihexoside, were identified. Concerning glucosinolates, only three compounds were identified, namely, glucoraphanin, glucoerucin, and dimeric 4-mercaptobutyl-glucosinolate (DMB). In some cases, the mass error for some glucosinolates was higher than 5 ppm; however, in those cases, the identification was confirmed by the use of standards.

In Table 2, we show the quantitative profile of the flavonols identified in Kyoh^®^ extract, highlighting the abundance of kaempferol-3,4′-O-diglucoside and quercetin-3,4′-O-diglucoside with 12 mg·g^−1^ and 5 mg·g^−1^, respectively. The concentrations of the rest of the identified flavonols went from 0.5 mg·g^−1^ to 2 mg·g^−1^, reaching a total concentration of flavonols in the sample of 21 mg·g^−1^. When considering the nature of the flavonol moiety of the glycosides, the most abundant concentrations were those constituted by kaempferol, followed by those with quercetin, while the leat abundant were those formed by isorhamnetin.

### 2.2. Evaluation of the Proliferative Capacity in Human Follicle Dermal Papilla Cells (HFDPC)

**Figure 1:**
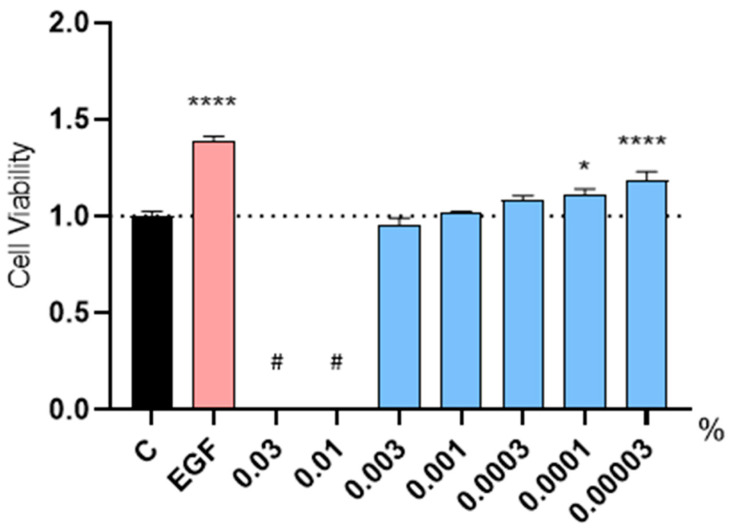
Graphical representation of the results showing the cell proliferation of human follicle dermal papilla cells after treatment with the tested products at the indicated dose range for 72 h (blue), including EGF at 20 ng/mL as a positive control (pink), compared to the non-treated control (black). Statistical significance is depicted as; * *p*-value < 0.05 and **** for *p* < 0.0001. # The product formed precipitates that interfered with the readout.

The effect of Kyoh^®^ extract on the cellular proliferation of HFDPC was evaluated using the MTT (3-(4,5-Dimethylthiazol-2-yl)-2,5-Diphenyltetrazolium Bromide) assay in low FBS (Fetal Bovine Serum) conditions. For the negative control, non-supplemented culture media was used, while for the positive control, culture media was supplemented with EGF (Epidermal Growth Factor) at 20 ng/mL. EGF increased cell proliferation compared to the non-treated control (Figure 1). This control result indicated that the proliferative potential of HFDPC remained intact, thus validating our experiment. We observed that high concentrations of the Kyoh^®^ extract (0.03% and 0.01%) interfered with the readout due to the color of the solution (Figure 1); therefore, these concentrations could not be evaluated. On the other hand, when Kyoh^®^ was used at 0.003%, 0.001%; or 0.0003%, no significant differences were found when compared to the non-treated control (Figure 1). Most importantly, treatment with Kyoh^®^ at 0.0001% or 0.00003% increased cell proliferation (Figure 1), demonstrating that Kyoh^®^ extract has proliferative activity in HFDPC.

### 2.3. Expression Analysis of Genes Involved in Hair Growth and Health in Human Follicle Dermal Papilla Cells

#### 2.3.1. 5α-Reductases

**Figure 2:**
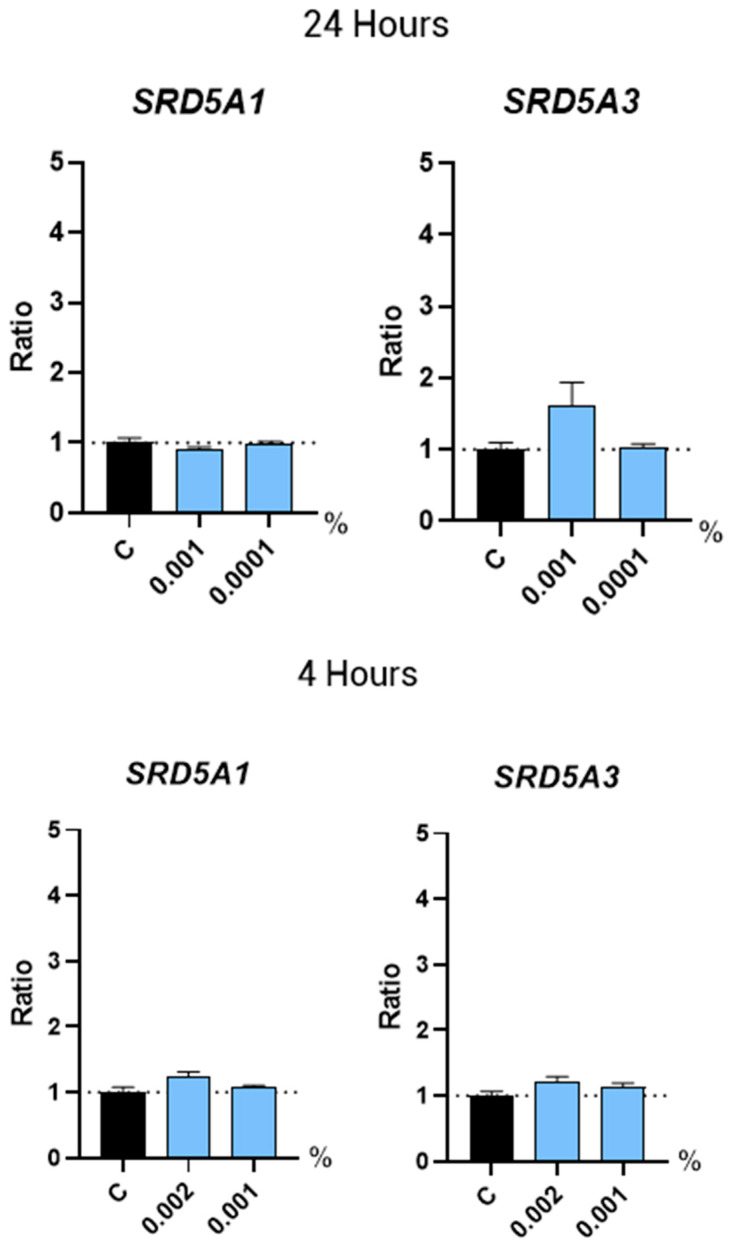
Bar graphs showing the gene expression levels of SRD5A1 and SRD5A3 in human follicle dermal papilla cells after treatment with Kyoh^®^ at the shown concentrations for 24 h (**top panels**) and 4 h (**bottom panels**), normalized to the non-treated control (C). Data are presented as mean ± standard error of the median (SEM).

5α-Reductases are enzymes that convert testosterone into the more potent androgen, DHT. Inhibition of 5-α-reductases to block formation of DHT is the molecular mechanism of action of the drugs Finasteride and Dutasteride [8,9,10]. Here, we analyzed the gene expression of the two main 5α-reductases associated with hair loss, SRD5A1 and SRD5A3. Non-supplemented culture media was used as a negative control for the gene expression studies. We found that treatment with Kyoh^®^ at the selected concentrations did not change the expression of these 5α-Reductases in HFDPC after 24 h of treatment, compared to the non-treated control (Figure 2). However, gene expression in humans could also be regulated at shorter periods of time. In order to check whether Kyoh^®^ induced a more immediate regulation of SRD5A1 andSRD5A3 gene expression in HFDPC, we performed another experiment to analyze their gene expression after 4 h of treatment. In this experiment, we included an experimental condition with a higher concentration of Kyoh^®^ (0.002%). Results showed that no changes were observed in SRD5A1 and SRD5A3 expression (Figure 2), indicating that Kyoh^®^ does not interfere with the DHT synthesis in the tested experimental conditions.

#### 2.3.2. Hair Growth Regulation by Growth Factors

**Figure 3:**
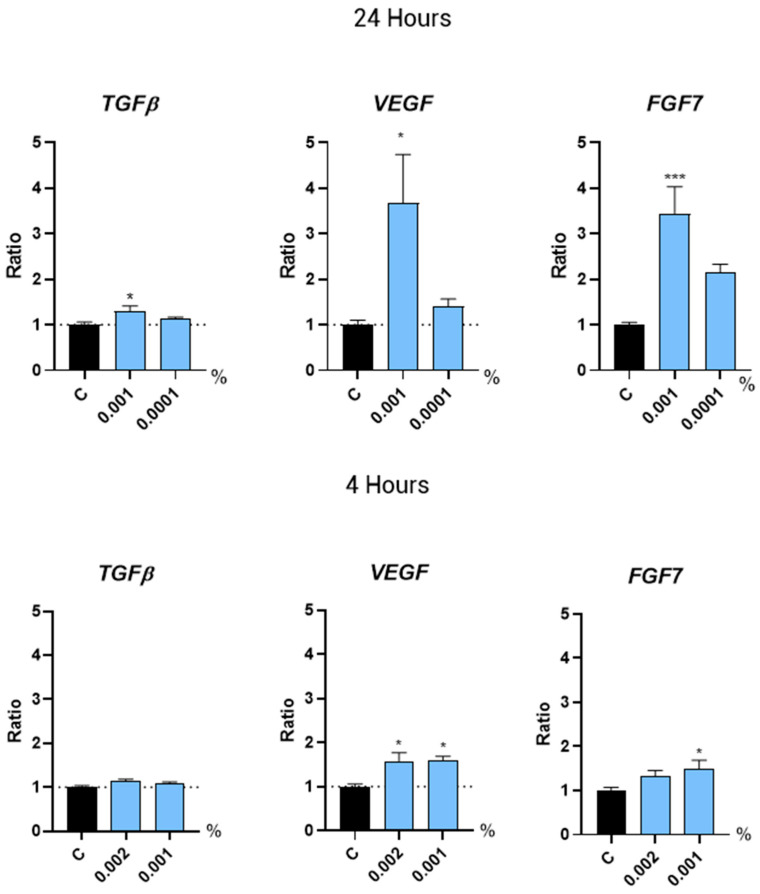
Bar graphs showing the gene expression levels of TGFB, VEGF, and FGF7 in human follicle dermal papilla cells after treatment with Kyoh^®^ at the shown concentrations for 24 h (**top panels**) and 4 h (**bottom panels**), normalized to the non-treated control (C). Data are presented as mean ± standard error of the median (SEM). Statistical significance is depicted as * *p*-value < 0.05 and *** *p*-value< 0.001.

**Figure 4:**
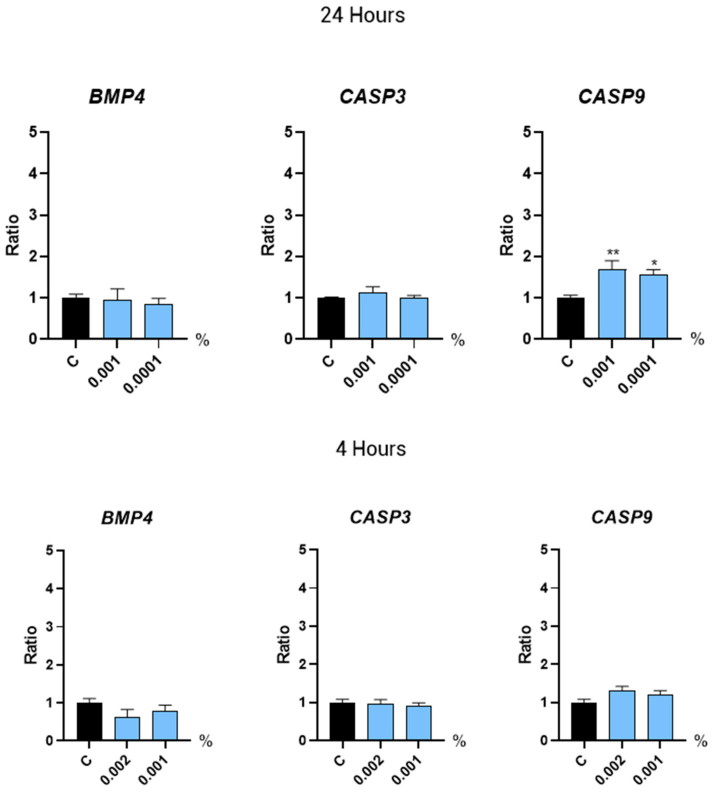
Bar graphs showing the gene expression levels of BMP4, CASP3, and CASP9 in human follicle dermal papilla cells after treatment with Kyoh^®^ at the shown concentrations for 24 h (**top panels**) and 4 h (**bottom panels**), normalized to the non-treated control (C). Data are presented as mean ± standard error of the median (SEM). Statistical significance is depicted as * *p*-value < 0.05 and ** *p*-value < 0.01.

Some growth factors stimulate hair growth and may counteract the effects of androgenetic alopecia by promoting follicle health and regeneration. Here, we examined the gene expression of several growth factors directly associated with hair growth. We observed that treatment with Kyoh^®^ at a concentration of 0.001% for 24 h induced the expression of the VEGF (+267.3%) and FGF7 (+244.3%) genes (Figure 3). Most importantly, we found that this concentration of Kyoh^®^ (0.001%) also induced gene expression of the growth factors VEGF and FGF7 (by 60.7% and 50.3%, respectively) at a shorter time point of 4 h (Figure 3). At 4 h, Kyoh^®^ did not induce TGFb expression, while at 24 h, the induction of TGFb was only 30% as compared to 267% and 244% for VEGF and FGF7, respectively. Also, another member of the TGF/BMP (Transforming Growth Factor/Bone Morphogenic Proteins) family of Growth factors, BMP4 (Bone Morphogenic Proteins 4), was not regulated by Kyoh^®^ at any of the time points tested (Figure 4). In addition, a higher concentration of Kyoh^®^ (0.002%) also increased the VEGF expression by 58.2% after 4 h of treatment (Figure 3), indicating that Kyoh^®^ regulates VEGF and FGF7 gene expression at 4h and 24 h, which are factors directly associated with hair growth in human follicle dermal papilla cells.

#### 2.3.3. Regulators of the Hair Growth Cycle

The hair growth cycle is influenced by a number of regulators that mediate the transition between phases, such as the BMP proteins and the caspases of the apoptotic programmed cell death pathway. In this study, the gene expression of BMP4, CASP3, and CASP9 was evaluated after a short (4 h) and a prolonged (24 h) treatment with Kyoh^®^. We observed that CASP9 expression increased after a 24 h treatment with Kyoh^®^ (Figure 4). However, the expression level of the rest of the analyzed genes and time points was not significantly different from the non-treated control (Figure 4).

#### 2.3.4. Oxidative Stress

**Figure 5:**
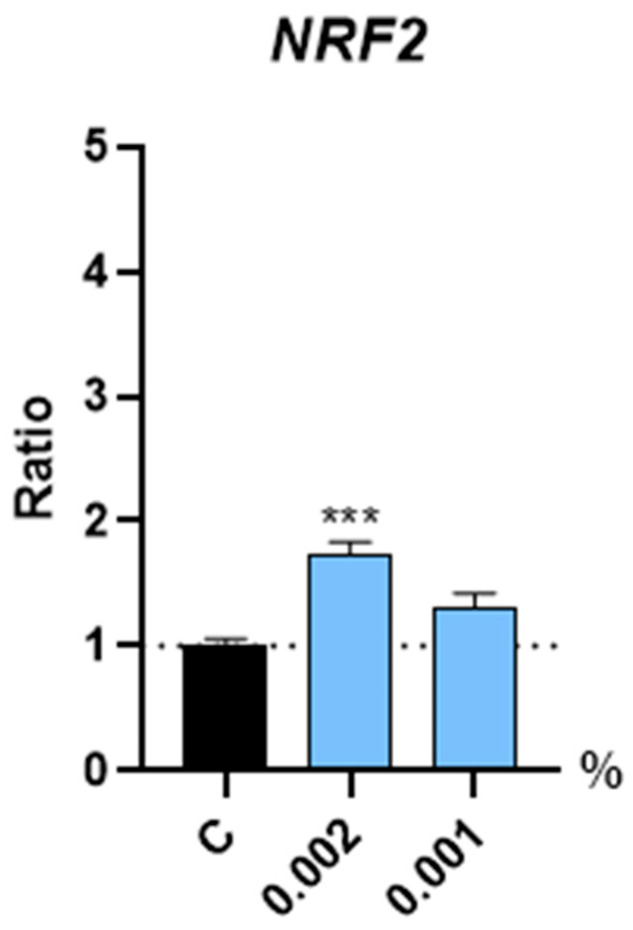
Bar graphs showing the gene expression levels of NRF2 in human follicle dermal papilla cells after treatment with Kyoh^®^ at the shown concentrations for 4 h, normalized to the non-treated control (C). Data are presented as mean ± standard error of the median (SEM). Statistical significance is depicted as *** *p*-value < 0.001.

Oxidative stress negatively impacts hair growth by damaging hair follicle cells, leading to impaired hair production and contributing to conditions like androgenetic alopecia. In this regard, NRF2 (Nuclear factor erythroid 2-related factor 2) plays a crucial role in hair growth by regulating antioxidant defenses and cellular protection mechanisms, which help to maintain the health and function of hair follicles. Our results showed that the expression of the NRF2 gene was induced upon a 4 h treatment with Kyoh^®^ at 0.002% by 71.2% (Figure 5), suggesting that HFDPCs are primed to respond to the oxidative stress conditions.

## 3. Discussion

The current treatments of androgenetic alopecia present several challenges. First, the efficacy of treatments, such as the FDA-approved minoxidil and finasteride, varies significantly among individuals and often requires long-term or permanent use to maintain hair growth, leading to potential side effects [12,32,33]. Additionally, the underlying genetic and hormonal factors driving androgenetic alopecia are complex and not yet fully understood, complicating the development of more targeted and effective therapies [12,32,33]. In this regard, plant extracts offer several advantages for the treatment of androgenetic alopecia, since they typically have fewer side effects compared to synthetic drugs, making them a safer option for long-term use, and their natural origin also appeals to individuals seeking organic treatment options [13].

In this study, we detected that the commercial extract (Kyoh^®^) had a bioactive compound composition characteristic of rocket leaves, with the presence of several flavonols glycosides, mostly, kaempferol, quercetin, and isorhamnetin mono- and diglucosides, and some of the most abundant glucosinolates previously described in rocket [26,34,35]. Considering the work of Martinez-Sanchez et al. [36] on the identification of the positions of these glycosides, along with subsequent studies that have confirmed their findings [26,34,35], we can more precisely identify these compounds as 3,4′-O-diglucosides and 3-O-glucosides. In addition, we also identified other flavonol glycosides, which have been less extensively reported in *E. sativa*, such as quercetin-3,3′,4′-O-triglucoside [34] and quercetin-3,4′-O-diglucoside [37]. Moreover, in this commercial extract, we identified, kaempferol-3-O-sophoroside and, tentatively, a second quercetin-trihexoside, both of which, to the best of our knowledge, have not been previously reported in rocket samples.

Regarding the quantitative profile of the studied extract, kaempferol-3,4′-O-diglucoside was found to be the most abundant flavonol with a concentration that fell in the concentration range found previously by Pasini et al. [35] for different *E. sativa* samples (8 to 24 mg·g^−1^). However, quercetin-3,4′-O-diglucoside, the second most abundant compound in Kyoh^®^, was found in a concentration ten times higher than previously reported (0.3 mg·g^−1^) [37], which could be explained by intraspecies variability and/or the effect of the extraction conditions employed. On the other hand, isorhamnetin-3,4′-O-diglucoside and total flavonol concentrations found in Kyoh^®^ were within the values found by Passini et al. [35], which were 1 to 5 mg·g^−1^ for isorhamnetin-3,4′-O-diglucoside and 10 to 31 mg·g^−1^ for total flavonols.

In scientific literature, some studies have proven the positive effect on capillary health of the bioactive compounds found in Kyoh^®^. For instance, Luo and Zhang [29] demonstrated that *Brassica oleracea* extract, rich in glucosinolates and sulforaphane, modulates the expression of cytokeratin genes, which are essential for hair shaft integrity and follicular function. Similarly, Ma et al. [31] reported that Camellia Seed Cake Extract, rich in kaempferol glycosides, regulates the expression of key growth factors, including VEGF, HGF, and IGF-1, thereby promoting hair follicle proliferation and maintenance. In our study, similar bioactive compounds were found to regulate the gene expression of VEGF, Nrf2, and FGF7, further supporting their role in modulating molecular pathways associated with hair growth and follicular health. In addition, we found that the evaluated extract shows promising potential in promoting hair growth and improving hair health by regulating HFDPC proliferation and VEGF and FGF7 regulation. Our finding showing that Kyoh^®^ promotes proliferation in human follicle dermal papilla cells is in line with other studies where different plant extracts have been reported to enhance the proliferation of this cell type in vitro [18,20,38,39,40,41,42]. Moreover, other plant extracts with a similar phytochemical composition, specifically an abundant presence of flavonols, have proved their activity in vivo; showing hair-growth-promoting activity in telogenic C57BL/6 N mice [30] and hair loss prevention in humans with androgenetic alopecia and telogen effluvium [24].

Interestingly, the dose of Kyoh^®^ proven in this study to enhance cell proliferation (1 µg/mL) is within the range or even lower than those doses described in the literature above, indicating that Kyoh^®^ has a strong proliferation-inducing effect compared to other plant extracts. This finding might be related to the potential antioxidant effect of Kyoh^®^ in human follicle dermal papilla cells, considering our result showing the increase in the expression of NRF2, a regulator that controls the basal and induced expression of an array of antioxidant response element-dependent genes to regulate the physiological and pathophysiological outcomes of oxidant exposure [43]. It is possible that the induction of the NRF2 gene could prime the cells to make them more resistant to cellular oxidative stress, hence enhancing cell proliferation, given the relationship established between the proliferative potential of the cells and oxidative stress [44,45,46].

The hair growth in the hair follicles undergoes life-long, cyclic transformations between the stages of active regeneration (anagen), apoptotic involution (catagen), and relative proliferative quiescence (telogen) [47]. Among the most studied androgens controlling the hair growth cycle is 5α-dihydrotestosterone (DHT), a hormone strongly associated with androgenetic alopecia [48]. There are three isoenzymes of 5α-reductase that catalyze the conversion of testosterone to DHT: steroid 5α-reductase type 1, 2, and 3 (SRD5A1, SRD5A2, and SRD5A3) [49,50,51]. Specifically, isoenzymes type 1 and 3 are highly present at pilosebaceous units in the papillae of individual hair follicles [52]. In this study, treatment with Kyoh^®^ did not alter the gene expression of SRD5A1 and SRD5A3 in the tested conditions, suggesting that its molecular mechanism is not based on the regulation of DHT synthesis. However, previous studies have indicated that plant extracts rich in kaempferol glycosides [31] or glucosinolates [29] prevent DHT-induced cell damage and apoptosis in dermal papilla cells. Taking into consideration that in the rocket extract evaluated in this study the same bioactive compounds are present, we could state that the same mechanism against DHT-induced cell damage could take place, though further experimentation is needed to substantiate this claim.

The cell growth cycle is regulated by different mechanisms, with apoptosis being one of the most relevant. Apoptosis contributes to the regulation of the hair cycle and growth by controlling the transition between the different phases of the hair growth cycle. Properly timed apoptosis ensures the renewal and regeneration of hair follicles, whereas dysregulated apoptosis can lead to hair loss and thinning [53]. In our study, we observed that Caspase-9 expression was induced after 24 h of treatment with Kyoh^®^. Caspases 1, 3, 8, and 9 are detected predominantly within the isthmic and infundibular hair follicle area for both normal and androgenetic alopecia patients, although the expression of some of these caspases may be higher in androgenetic alopecia patients [53]. Importantly, our proliferation assay revealed that the used doses of Kyoh^®^ are not cytotoxic for the cells; indeed, one of them (0.0001%) has a proliferative effect, suggesting that the detected overexpression of Caspase-9 is associated with a regulatory effect rather than a cytotoxic effect in the treated cells. In the context of the regulation of the hair growth cycle, the growth and differentiation of hair follicles are also controlled by reciprocal interactions between the dermal papilla and the surrounding epidermal hair precursors including bone morphogenetic proteins (BMPs) [54]. In particular, it has been shown that the BMP2 and BMP4 genes decrease hair growth by inhibiting the telogen–anagen transition [54]. In this study, we found that Kyoh^®^ does not have any effect on the transcription of BMP4, overall suggesting that Kyoh^®^ is not associated with the regulation of the transitions between the different phases of the hair growth cycle. The lack of significant changes in the expression of CASP3, CASP9, BMP4, SRD5A1, SRD5A2, and SRD5A3 suggests that the tested bioactive compounds primarily influence pathways related to angiogenesis, follicular proliferation, and oxidative stress (via VEGF, FGF7, and Nrf2) rather than apoptosis (CASP3, CASP9), morphogenesis (BMP4), or androgen metabolism (SRD5A family). This specificity may be due to the compounds’ selective activation of signaling pathways involved in hair follicle maintenance rather than broad systemic effects.

Growth factors are one of the cornerstones of the regulation and health of hair growth [55,56,57,58]. The vascular endothelial growth factor (VEGF) is considered the most important mediator for the process of angiogenesis involved in hair growth development and it has been shown that upregulation of VEGF expression induces hair growth [59]. On the other side, fibroblast growth factor 7 (FGF7) plays a crucial role in stimulating hair matrix cell proliferation and hair growth by prolonging the anagen phase, being an important regulator of the hair growth cycle [60,61,62,63]. It is noteworthy that other growth factors, such as transforming growth factor beta (TGBb), are associated with hair loss and the progression of androgenetic alopecia [64,65,66,67,68]. Our results showed a slight increase in TGFB expression, which may be due to the cascade signaling effects after 24 h, as Kyoh^®^ extract treatment does not induce TGFb expression or BMP4 (from the same family of proteins as TGFB) expression after 4 h. Most importantly, our results demonstrated that treatment with Kyoh^®^ activated the gene expression of VEGF and FGF7 both at short term (4 h treatment) and after 24 h. Interestingly, this molecular mechanism resembles that described for Minoxidil, which is based on the enhancement of the expression of the VEGF gene [69]. Therefore, our results show that Kyoh^®^ has a quick and sustained effect in the induction of growth factors required for hair growth and health and might be a natural alternative to currently approved chemicals such as Minoxidil since it shares the same molecular targets.

## 4. Materials and Methods

### 4.1. Cell Culture

In this study, human follicle dermal papilla cells (HFDPCs) (Promocell, Heidelberg, Germany) were used. Cells were maintained in HFDPC-specific media (Promocell) supplemented with 10% fetal bovine serum (FBS; Gibco, Life Technologies, Carlsbad, CA, USA), 1% penicillin–streptomycin (Gibco, Life Technologies), and 1% L-glutamine (Gibco, Life Technologies). HFDPCs were incubated at 37 °C in a humidified atmosphere containing 5% CO_2_. The medium was replaced every 2–3 days, and the cells were passaged at approximately 80% confluency using trypsin-EDTA (0.05%; Sigma-Aldrich, St. Louis, MO, USA) for detachment.

### 4.2. Reagents and Standards

The following reagents were used: distilled Water (Braun), cell-specific culture medium and supplements for HFDPC (PromoCell), PBS (Gibco), Trypan Blue (Bio-Rad, Hercules, CA, USA), Trypsin (Sigma-Aldrich), Dimethylsulfoxide (DMSO, Sigma-Aldrich), MTT reagent [3-(4,5-Dimethylthiazol-2-yl)-2,5-Diphenyltetrazolium Bromide] (Invitrogen, Waltham, MA, USA), Epidermal growth factor (EGF, Sigma-Aldrich), RNeasy extraction kit (Qiagen, Hilden, Germany), DNAse-I (Qiagen), PrimeScript RT reagent kit (Perfect Real Time; TaKaRa, Shiga, Japan), oligonucleotides for qPCR amplification TaqMan^®^ for qRT-PCR (IDT, San Jose, CA, USA). Analytical standards of glucoerucin, rutin, kaempferol-3-O-glucoside, and quercetin-3-O-glucoside were acquired at Phytolab (Vestenbergsgreuth, Germany), kaempferol was acquired at Sigma Aldrich, glucoraphanin was acquired at Extrasynthese (Genay, France), and kaempferol-3-O-sophoroside was acquired at Biopurify (Sichuan, China).

### 4.3. Sample and Sample Preparation

The sample evaluated in this study was a dried commercial powdered extract of rocket, branded as Kyoh^®^ and standardized in more than 1.5% (*w*/*w*) erucosides^®^, as the sum of the bioactive compounds present in the product. This extract is obtained from Eruca sativa dry leaves in a hydroethanolic solvent, through a proprietary extraction and manufacturing process registered as ActiveNature^TM^ Tech. The sample was provided and produced by the company Pharmactive Biotech Products S.L.U., in their own manufacturing facilities (Madrid, Spain).

Regarding sample preparation for HPLC analysis, 100 mg of rocket extract was redissolved in 5 mL of methanol/water (50:50, *v*/*v*) using an ultrasonic bath (GT Sonic, Shenzhen, China) at around 30 °C for 10 min. Samples were then filtered through 0.22 µm nylon filters (Agilent, Santa Clara, CA, USA) and immediately stored at −4 °C until analyses were carried out.

### 4.4. HPLC-DAD-HRMS Characterization

The system employed for the characterization of the sample was an Agilent 1260 Infinity II Prime LC System with a diode array detector (DAD) coupled to a 6230B time-of-flight (ToF) MS detector provided with an Agilent Jet Stream Electrospray Ionization (AJS-ESI) source (Agilent Technologies, Santa Clara, CA, USA). Analysis was carried out in a Poroshell 120 EC-C18 column (150 × 3.0 mm, 2.7 µm; Agilent) at 30 °C using water (eluent A) and acetonitrile (eluent B), both with 0.01% formic acid, as mobile phase, and following this gradient: 5% B (0 min), 5% B (1 min), 25% B (13 min), 95% B (20 min), and 95% B (22 min) at a 0.5 mL/min flow. DAD spectrum was acquired between 190 and 700 nm, while MS was acquired between 100 and 1700 m/z at negative polarity. AJS-ESI ionization parameters were 300 °C for drying gas temperature, 13 L/min for drying gas flow, 35 psi for nebulizer, 250 °C for sheat gas temperature, 11 L/min for sheat gas flow, 500 V for nozzle voltage, and 3.5 kV for capillary voltage.

Compound identification was based on UV spectra, chromatographic retention, and exact mass spectrometry data, which was confirmed, when possible, by co-injection of the corresponding commercial standards (Section 4.2). The identifications were considered tentative when analytical standards were not available. For quantitative analysis, absorbance was measured at 340 nm and an external standard calibration curve of rutin (5–200 ppm) was used for all the flavonols. Data acquisition and processing were performed using MassHunter Software (v.10.1.48, Agilent Technologies).

### 4.5. Cell Proliferation Assay

Cell numbers and viability were determined using Trypan-Blue staining and counting in a Bürker chamber under the microscope. HFDPCs were cultured overnight at a density of 4.000 cells/well in a 96-well plate in supplemented growth medium; 24 h later, the culture medium was replaced with fresh medium containing 0.5% supplement, and the products were tested at 7 different concentrations: 0.03, 0.01, 0.003, 0.001, 0.0003, 0.0001, and 0.00003%. In parallel, cells were cultured with EGF (20 ng/mL) as a positive control for proliferation-promoting compounds. After 72 h of incubation, the medium was removed and MTT solution was added to each well. Plates were incubated at 37 °C for 3 h. MTT reagent was discarded, and DMSO at 100% was added to each well to solubilize formazan crystals, then the absorbance was measured at 550 nm and 620 nm as a reference on a scanning multi-well spectrophotometer.

### 4.6. Analysis of Gene Expression by qRT-PCR

For the gene expression assay, HFDPC were cultured in supplemented growth medium at a density of 100,000 cells/well in 12-well plates at 37 °C, 5% CO_2_; 24 h later, the medium was replaced with fresh medium containing the products at the selected doses and incubated for 4 h or 24 h at 37 °C, 5% CO_2_. Afterward, cells were collected in a lysis buffer to proceed with the RNA extraction. Total RNA was extracted using an RNeasy kit (Qiagen) and treated with DNAseI to remove any contamination from genomic DNA. RNA quality and quantity were checked in a Nano-Drop spectrophotometer, and 1 µg of total RNA was used to synthesize cDNA, using a First-strand Synthesis kit (TaKaRa). Finally, quantitative PCR (qPCR) was performed in a real-time PCR machine (QuantStudio 5, Applied BioSystem, MA, USA). To perform raw data analysis, we used the 2–∆∆Ct method [70] to calculate the gene relative expression ratio to non-treated control (C). Actin (ACT) was used as a reference housekeeping gene.

### 4.7. Statistical Analysis

Statistical analysis was performed with GraphPad Prism software (v. 10.1.1.). The MTT assay was set with eight replicates per condition and the qRT-PCR assay was set with five replicates per condition. All collected data were subjected to the normality test using the Shapiro–Wilk method. Data outliers were identified with the ROUT method (Q = 5%) and excluded from the analysis if found. Data were statistically analyzed by one-way ANOVA test and Dunnet’s post hoc multiple comparisons test. Statistical significance was declared at *p* < 0.05, 95% of confidence. Bars in the charts represent the mean value for each condition and error bars indicate the standard error of the mean (SEM) for each group of values.

## 5. Conclusions

Plant extracts have emerged as promising treatments for androgenetic alopecia due to their natural bioactive compounds. Most of the described plant extracts have one single in vitro molecular target; however, here we provide evidence that rocket targets different molecular processes associated with hair growth and health (HFDPC proliferation and VEGF, FGF7, and NRF2 expression). In this study, we show that flavonol-rich rocket extract (Kyoh^®^) has a promising effect on promoting hair growth, evidenced by its proliferation-enhancing activity, potential antioxidant priming, and stimulatory effect on growth factors associated with hair growth.

## Figures and Tables

**Table 1:** Bioactive compounds identified in rocket extract by HPLC-DAD-TOF.

Compound	Rt (min)	Molecular Formula	MW	Detected *m*/*z* Ions (Fragments)	Mass Error (ppm)	Standard
Flavonols						
Quercetin-3,3′,4′-O-triglucoside	6.12	C_33_H_40_O_22_	788.2024	787.1946 [M-H]^−^ (625.1521 [M-H-Glu]^−^)	1.6	No
Quercetin-trihexoside	11.1	C_33_H_40_O_22_	788.2024	787.1946 [M-H]^−^ (625.1521 [M-H-Glu]^−^ 463.1939 [M-H-2Glu]^−^)	1.6	No
Quercetin-3,4′-O-diglucoside	11.4	C_27_H_30_O_17_	626.1483	625.1428 [M-H]^−^ 671.1604 [M+COO]^−^ (463.1064 [M-H-Glu]^−^)	2.6	No
Kaempferol-3,4′-O-diglucoside	11.5	C_27_H_30_O_16_	610.1534	609.1474 [M-H]^−^	2.4	No
Isorhamnetin-3,4′-O-diglucoside	12.0	C_28_H_32_O_17_	640.1639	639.1582 [M-H]^−^	2.6	No
Kaempferol-3-O-sophoroside	12.1	C_27_H_30_O_16_	610.1534	609.1481 [M-H]^−^ (447.1616 [M-H-Glu]^−^)	4.1	Yes
Rutin	12.7	C_27_H_30_O_16_	610.1534	609.1481 [M-H]^−^ (447.1784 [M-H-Glu]^−^)	4.4	Yes
Quercetin-3-O-glucoside	13.5	C_21_H_20_O_12_	464.0955	463.0896 [M-H]^−^	3.7	No
Kaempferol-3-O-glucoside	14.8	C_21_H_20_O_11_	448.1006	447.0947 [M-H]^−^	3.2	Yes
Isorhamnetin-3-O-glucoside	15.2	C_22_H_22_O_12_	478.1127	477.1050 [M-H]^−^	3.2	No
Kaempferol	17.2	C_15_H_10_O_6_	286.0477	285.0419 [M-H]^−^ 345.0654 [M+COO]^−^	5.0	Yes
Glucosinolates						
Glucoraphanin	1.7	C_12_H_23_O_10_S_3_	437.0484	436.0368 [M-H]^−^	−5.2	Yes
Glucoerucin	5.4	C_12_H_23_NO_9_S_3_	421.0535	420.0488 [M-H]^−^	7.3	Yes
DMB	6.5	C_22_H_40_N_2_O_18_S_6_	812.0600	811.0553 [M-H]^−^	4.5	No

Glu: Glucose (C_6_H_10_O_5_: 162.0528 *m*/*z*).

**Table 2:** Flavonols concentration in Kyoh^®^ extract.

Compound	mg·g^−1^ Dry Weight
Quercetin-3,3′,4′-O-triglucoside	0.88 (0.06) *
Quercetin-trihexoside	0.47 (0.01)
Quercetin-3,4′-O-diglucoside	5.391 (0.002)
Kaempferol-3,4′-O-diglucoside	11.997 (0.005)
Isorhamnetin-3,4′-O-diglucoside	1.66 (0.01)
Kaempferol-3-O-sophoroside	0.51 (0.01)
Rutin	0.373 (0.001)
Quercetin-3-O-glucoside	0.704 (0.008)
Kaempferol-3-O-glucoside	1.522 (0.002)
Isorhamnetin-3-O-glucoside	0.563 (0.002)
Kaempferol	<LOQ
Total flavonols	21.2 (0.04)

* Standard deviation in parenthesis (*n* = 2).

## Data Availability

Data are contained within the article or Supplementary Material.

## Supplementary Materials

The following supporting information can be downloaded at: www.mdpi.com/article/10.3390/molecules30071489/s1, Figure S1: UV spectra of most abundant flavonols identified in Kyoh® extract.

## Abbreviations

The following abbreviations are used in this manuscript:

ACT	Actin
ANOVA	Analysis of Variance
BMP	Bone Morphogenic Proteins
CASP3	Caspase 3
CASP9	Caspase 9
DAD	Diode Array Detector
DHT	Hormone Dihydrotestosterone
DMB	Dimeric 4-mercaptobutyl-glucosinolate
DMSO	Dimethylsulfoxide
EDTA	Ethylenediaminetetraacetic acid
EGF	Epidermal Growth factor
FBS	Fetal Bovine Serum
FDA	Food and Drug Administration
FGF7	Fibroblast Growth Factor 7
HFDPC	Human Follicle Dermal Papilla Cells
HPLC-DAD-MS	High-Performance Liquid Chromatography–Diode Array Detector–Mass Spectrometry
HPLC-DAD-HRMS	High-Performance Liquid Chromatography–Diode Array Detector–High-Resolution Mass Spectrometry
MTT	3-(4,5-Dimethylthiazol-2-yl)-2,5-Diphenyltetrazolium Bromide
MW	Molecular Weight
NRF2	Nuclear factor erythroid 2-related factor 2
ROUT	Robust Regression and Outliner Detection
RT-PCR	Reverse Transcription Polymerase Chain Reaction
SEM	Standard Error of Mean
SRD5A1	Steroid 5 alpha-reductase 1
SRD5A2	Steroid 5 alpha-reductase 1
SRD5A3	Steroid 5 alpha-reductase 3
TGBb	Transforming Growth Factor beta
TGF	Transforming Growth Factor
VEGF	Vascular Endothelial Growth Factor

## References

[1] McElwee K.J., Shapiro J.S. (2012). Promising therapies for treating and/or preventing androgenic alopecia. Skin Ther. Lett..

[2] Martinez-Jacobo L., Villarreal-Villarreal C.D., Ortiz-López R., Ocampo-Candiani J., Rojas-Martínez A. (2018). Genetic and molecular aspects of androgenetic alopecia. Indian J. Dermatol. Venereol. Leprol..

[3] Ntshingila S., Oputu O., Arowolo A.T., Khumalo N.P. (2023). Androgenetic alopecia: An update. JAAD Int..

[4] Owecka B., Tomaszewska A., Dobrzeniecki K., Owecki M. (2024). The Hormonal Background of Hair Loss in Non-Scarring Alopecias. Biomedicines.

[5] Liang B., Yang C., Zuo X., Li Y., Ding Y., Sheng Y., Zhou F., Cheng H., Zheng X., Chen G. (2013). Genetic variants at 20p11 confer risk to androgenetic alopecia in the Chinese Han population. PLoS ONE.

[6] Rinaldi S., Bussa M., Mascaro A. (2016). Update on the treatment of androgenetic alopecia. Eur. Rev. Med. Pharmacol. Sci..

[7] Messenger A.G., Rundegren J. (2004). Minoxidil: Mechanisms of action on hair growth. Br. J. Dermatol..

[8] Mazzarella G., Loconsole G., Cammisa G., Mastrolonardo G., Vena G. (1997). Topical finasteride in the treatment of androgenic alopecia. Preliminary evaluations after a 16-month therapy course. J. Dermatol. Treat..

[9] Piraccini B.M., Blume-Peytavi U., Scarci F., Jansat J.M., Falqués M., Otero R., Tamarit M.L., Galván J., Tebbs V., Massana E. (2022). Efficacy and safety of topical finasteride spray solution for male androgenetic alopecia: A phase III, randomized, controlled clinical trial. J. Eur. Acad. Dermatol. Venereol. JEADV.

[10] Arif T., Dorjay K., Adil M., Sami M. (2017). Dutasteride in Androgenetic Alopecia: An Update. Curr. Clin. Pharmacol..

[11] Blume-Peytavi U., Lönnfors S., Hillmann K., Garcia Bartels N. (2012). A randomized double-blind placebo-controlled pilot study to assess the efficacy of a 24-week topical treatment by latanoprost 0.1% on hair growth and pigmentation in healthy volunteers with androgenetic alopecia. J. Am. Acad. Dermatol..

[12] Devjani S., Ezemma O., Kelley K.J., Stratton E., Senna M. (2023). Androgenetic Alopecia: Therapy Update. Drugs.

[13] Choi J.Y., Boo M.Y., Boo Y.C. (2024). Can Plant Extracts Help Prevent Hair Loss or Promote Hair Growth? A Review Comparing Their Therapeutic Efficacies, Phytochemical Components, and Modulatory Targets. Molecules.

[14] Baek J.H., Lee S.Y., Yoo M., Park W.S., Lee S.J., Boo Y.C., Koh J.S. (2011). Effects of a new mild shampoo for preventing hair loss in Asian by a simple hand-held phototrichogram technique. Int. J. Cosmet. Sci..

[15] Choi H.C., Nam G.W., Jeong N.H., Choi B.Y. (2019). Hair Growth Promotion by Extracts of Inula Helenium and Caesalpinia Sappan Bark in Patients with Androgenetic Alopecia: A Pre-clinical Study Using Phototrichogram Analysis. Cosmetics.

[16] Kim B.H., Lee W.Y., Trinh T.A., Pyo J.S., Lee S., Kim C.E., Lee D.H., Park E.S., Kang K.S. (2020). Hair Growth Effect of Emulsion Extracted Brevilin A, a JAK3 Inhibitor, from *Centipeda minima*. Processes.

[17] Hashimoto M., Kawai Y., Masutani T., Tanaka K., Ito K., Iddamalgoda A. (2022). Effects of watercress extract fraction on R-spondin 1-mediated growth of human hair. Int. J. Cosmet. Sci..

[18] Liang C.H., Lin Y.H., Lin Y.K., Chiang C.F. (2023). Hair growth-promotion effects and antioxidant activity of the banana flower extract HappyAngel^®^: Double-blind, placebo-controlled trial. Food Sci. Hum. Wellness.

[19] Ham S., Lee Y.I., Kim I.A., Suk J., Jung I., Jeong J., Lee J.H. (2023). Efficacy and safety of persimmon leaf formulated with green tea and sophora fruit extracts (BLH308) on hair growth: A randomized, double-blind, placebo-controlled clinical trial. Skin Res. Technol..

[20] You J., Woo J., Roh K., Jeon K., Jang Y., Choi S., Ryu D., Cho E., Park D., Lee J. (2024). Evaluation of efficacy of *Silybum marianum* flower extract on the mitigating hair loss in vitro and in vivo. J. Cosmet. Dermatol..

[21] Vicente R., Leite ESilva V., Baby A., Velasco M., Bedin V. (2009). Double-blind, randomized, placebo-controlled trial of a cream containing the *Stryphnodendron adstringens* (Martius) Coville bark extract for suppressing terminal hair growth. J. Eur. Acad. Dermatol. Venereol..

[22] Choi J.S., Park J.B., Moon W.S., Moon J.N., Son S.W., Kim M.R. (2015). Safety and Efficacy of Rice Bran Supercritical CO_2_ Extract for Hair Growth in Androgenic Alopecia: A 16-Week Double-Blind Randomized Controlled Trial. Biol. Pharm. Bull..

[23] Yu J.Y., Gupta B., Park H.G., Son M., Jun J.-H., Yong C.S., Kim J.A., Kim J.O. (2017). Preclinical and Clinical Studies Demonstrate That the Proprietary Herbal Extract DA-5512 Effectively Stimulates Hair Growth and Promotes Hair Health. Evid. Based Complement. Altern. Med..

[24] Pekmezci E., Dündar C., Türkoğlu M. (2018). A proprietary herbal extract against hair loss in androgenetic alopecia and telogen effluvium: A placebo-controlled, single-blind, clinical-instrumental study. Acta Dermatovenerol. Alp. Panon. Adriat..

[25] Abd-Elsalam R.M., El Badawy S.A., Ogaly H.A., Ibrahim F.M., Farag O.M., Ahmed K.A. (2021). Eruca sativa seed extract modulates oxidative stress and apoptosis and up-regulates the expression of Bcl-2 and Bax genes in acrylamide-induced testicular dysfunction in rats. Environ. Sci. Pollut. Res. Int..

[26] Bell L., Wagstaff C. (2019). Rocket science: A review of phytochemical & health-related research in *Eruca* & *Diplotaxis* species. Food Chem. X.

[27] Garg G., Sharma V. (2014). *Eruca sativa* (L.): Botanical Description, Crop Improvement, and Medicinal Properties. J. Herbs Spices Med. Plants.

[28] Shatalebi M.A., Safaeian L., Baradaran A., Alamdarian M. (2016). Preparation and evaluation of a hair wax containing propolis and Eruca sativa seed oil for hair growth. Adv. Biomed. Res..

[29] Luo Z., Zhang X. (2022). Brassica oleracea extract, glucosinlates, and sulforaphane promote hair growth in vitro and ex vivo. J. Cosmet. Dermatol..

[30] Zhang N.N., Park D.K., Park H.J. (2013). Hair growth-promoting activity of hot water extract of *Thuja orientalis*. BMC Complement. Altern. Med..

[31] Ma L., Shen H., Fang C., Chen T., Wang J. (2022). Camellia Seed Cake Extract Supports Hair Growth by Abrogating the Effect of Dihydrotestosterone in Cultured Human Dermal Papilla Cells. Molecules.

[32] Nestor M.S., Ablon G., Gade A., Han H., Fischer D.L. (2021). Treatment options for androgenetic alopecia: Efficacy, side effects, compliance, financial considerations, and ethics. J. Cosmet. Dermatol..

[33] Kaiser M., Abdin R., Gaumond S.I., Issa N.T., Jimenez J.J. (2023). Treatment of Androgenetic Alopecia: Current Guidance and Unmet Needs. Clin. Cosmet. Investig. Dermatol..

[34] Bell L., Oruna-Concha M.J., Wagstaff C. (2015). Identification and quantification of glucosinolate and flavonol compounds in rocket salad (*Eruca sativa*, *Eruca vesicaria* and *Diplotaxis tenuifolia*) by LC–MS: Highlighting the potential for improving nutritional value of rocket crops. Food Chem..

[35] Pasini F., Verardo V., Caboni M.F., D’Antuono L.F. (2012). Determination of glucosinolates and phenolic compounds in rocket salad by HPLC-DAD–MS: Evaluation of *Eruca sativa* Mill. and *Diplotaxis tenuifolia* L. genetic resources. Food Chem..

[36] Martinez-Sánchez A., Llorach R., Gil M.I., Ferreres F. (2007). Identification of New Flavonoid Glycosides and Flavonoid Profiles to Characterize Rocket Leafy Salads (*Eruca vesicaria* and *Diplotaxis tenuifolia*). J. Agric. Food Chem..

[37] Sut S., Boschiero I., Solana M., Malagoli M., Bertucco A., Dall’Acqua S. (2018). Supercritical CO_2_ Extraction of *Eruca sativa* Using Cosolvents: Phytochemical Composition by LC-MS Analysis. Molecules.

[38] Junlatat J., Sripanidkulchai B. (2014). Hair Growth-Promoting Effect of *Carthamus tinctorius* Floret Extract. Phytother. Res..

[39] Lee H., Kim N.H., Yang H., Bae S.K., Heo Y., Choudhary I., Kwon Y.C., Byun J.K., Yim H.J., Noh B.S. (2016). The Hair Growth-Promoting Effect of *Rumex japonicus* Houtt. Extract. Evid.-Based Complement. Altern. Med. ECAM.

[40] Wen T.C., Li Y.S., Rajamani K., Harn H.J., Lin S.Z., Chiou T.W. (2018). Effect of *Cinnamomum osmophloeum* Kanehira Leaf Aqueous Extract on Dermal Papilla Cell Proliferation and Hair Growth. Cell Transplant..

[41] Serruya R., Maor Y. (2021). Hair growth-promotion effects at the cellular level and antioxidant activity of the plant-based extract Phyllotex^TM^. Heliyon.

[42] Wang J., Shen H., Chen T., Ma L. (2022). Hair growth-promoting effects of Camellia seed cake extract in human dermal papilla cells and C57BL/6 mice. J. Cosmet. Dermatol..

[43] Ma Q. (2013). Role of nrf2 in oxidative stress and toxicity. Annu. Rev. Pharmacol. Toxicol..

[44] Immenschuh S., Baumgart-Vogt E. (2005). Peroxiredoxins, oxidative stress, and cell proliferation. Antioxid. Redox Signal..

[45] Kadam S.D., Gucek M., Cole R.N., Watkins P.A., Comi A.M. (2012). Cell proliferation and oxidative stress pathways are modified in fibroblasts from Sturge-Weber syndrome patients. Arch. Dermatol. Res..

[46] Diaz-Vivancos P., de Simone A., Kiddle G., Foyer C.H. (2015). Glutathione–linking cell proliferation to oxidative stress. Free Radic. Biol. Med..

[47] Geyfman M., Plikus M.V., Treffeisen E., Andersen B., Paus R. (2015). Resting no more: Re-defining telogen, the maintenance stage of the hair growth cycle: Resting stage of the hair follicle cycle. Biol. Rev..

[48] Rastegar H., Ashtiani H.A., Aghaei M., Barikbin B., Ehsani A. (2015). Herbal Extracts Induce Dermal Papilla Cell Proliferation of Human Hair Follicles. Ann. Dermatol..

[49] Yamana K., Labrie F., Luu-The V. (2010). Human type 3 5α-reductase is expressed in peripheral tissues at higher levels than types 1 and 2 and its activity is potently inhibited by finasteride and dutasteride. Horm. Mol. Biol. Clin. Investig..

[50] Russell D.W., Wilson J.D. (1994). Steroid 5 alpha-reductase: Two genes/two enzymes. Annu. Rev. Biochem..

[51] Agis-Balboa R.C., Pinna G., Zhubi A., Maloku E., Veldic M., Costa E., Guidotti A. (2006). Characterization of brain neurons that express enzymes mediating neurosteroid biosynthesis. Proc. Natl. Acad. Sci. USA.

[52] Bernard B.A. (1994). Molecular approach of hair biology. C. R. Seances Soc. Biol. Fil..

[53] Wang W., Wang H., Long Y., Li Z., Li J. (2023). Controlling Hair Loss by Regulating Apoptosis in Hair Follicles: A Comprehensive Overview. Biomolecules.

[54] Kulessa H., Turk G., Hogan B.L. (2000). Inhibition of Bmp signaling affects growth and differentiation in the anagen hair follicle. EMBO J..

[55] Ozeki M., Tabata Y. (2002). Promoted growth of murine hair follicles through controlled release of vascular endothelial growth factor. Biomaterials.

[56] Alexandrescu D.T., Kauffman C.L., Dasanu C.A. (2009). The cutaneous epidermal growth factor network: Can it be translated clinically to stimulate hair growth?. Dermatol. Online J..

[57] Zhao J., Harada N., Okajima K. (2011). Dihydrotestosterone inhibits hair growth in mice by inhibiting insulin-like growth factor-I production in dermal papillae. Growth Horm. IGF Res..

[58] Yun Y.R., Won J.E., Jeon E., Lee S., Kang W., Jo H., Jang J.H., Shin U.S., Kim H.W. (2010). Fibroblast growth factors: Biology, function, and application for tissue regeneration. J. Tissue Eng..

[59] Lee C.Y., Su C.H., Chiang C.Y., Wu C.N., Kuan Y.H. (2021). Observation of the Expression of Vascular Endothelial Growth Factor and the Potential Effect of Promoting Hair Growth Treated with Chinese Herbal BeauTop. Evid.-Based Complement. Altern. Med. ECAM.

[60] Rosenquist T.A., Martin G.R. (1996). Fibroblast growth factor signalling in the hair growth cycle: Expression of the fibroblast growth factor receptor and ligand genes in the murine hair follicle. Dev. Dyn..

[61] Bae W.Y., Jung W.H., Shin S.L., Kim T.R., Sohn M., Suk J., Jung I., Lee Y.I., Lee J.H. (2024). Heat-treated *Limosilactobacillus fermentum* LM1020 with menthol, salicylic acid, and panthenol promotes hair growth and regulates hair scalp microbiome balance in androgenetic alopecia: A double-blind, randomized and placebo-controlled clinical trial. J. Cosmet. Dermatol..

[62] Alessandrini A.M., Bruni F., Piraccini B.M., Starace M. (2021). The Effectiveness and Tolerability of Preformed Growth Factors Vehiculated Through Iontophoresis on Patients with Androgenetic Alopecia and Telogen Effluvium: A Clinical Study. Dermatol. Pract. Concept..

[63] Gentile P., Garcovich S. (2019). Advances in Regenerative Stem Cell Therapy in Androgenic Alopecia and Hair Loss: Wnt pathway, Growth-Factor, and Mesenchymal Stem Cell Signaling Impact Analysis on Cell Growth and Hair Follicle Development. Cells.

[64] Shin H., Yoo H.G., Inui S., Itami S., Kim I.G., Cho A.-R., Lee D.H., Park W.S., Kwon O., Cho K.H. (2013). Induction of transforming growth factor-beta 1 by androgen is mediated by reactive oxygen species in hair follicle dermal papilla cells. BMB Rep..

[65] Inui S., Fukuzato Y., Nakajima T., Yoshikawa K., Itami S. (2002). Androgen-inducible TGF-beta1 from balding dermal papilla cells inhibits epithelial cell growth: A clue to understand paradoxical effects of androgen on human hair growth. FASEB J..

[66] Liang Y., Tang X., Zhang X., Cao C., Yu M., Wan M. (2023). Adipose Mesenchymal Stromal Cell-Derived Exosomes Carrying MiR-122-5p Antagonize the Inhibitory Effect of Dihydrotestosterone on Hair Follicles by Targeting the TGF-β1/SMAD3 Signaling Pathway. Int. J. Mol. Sci..

[67] Inui S., Fukuzato Y., Nakajima T., Yoshikawa K., Itami S. (2003). Identification of androgen-inducible TGF-beta1 derived from dermal papilla cells as a key mediator in androgenetic alopecia. J. Investig. Dermatol. Symp. Proc..

[68] Naruse T., Aoki M., Fujimoto N., Arase S., Oura H., Ueda Y., Ikeda A. (2017). Novel ALK5 inhibitor TP0427736 reduces TGF-β induced growth inhibition in human outer root sheath cells and elongates anagen phase in mouse hair follicles. Pharmacol. Rep. PR.

[69] Yum S., Jeong S., Kim D., Lee S., Kim W., Yoo J.W., Kim J.-A., Kwon O.S., Kim D.-D., Min D.S. (2017). Minoxidil Induction of VEGF Is Mediated by Inhibition of HIF-Prolyl Hydroxylase. Int. J. Mol. Sci..

[70] Livak K.J., Schmittgen T.D. (2001). Analysis of Relative Gene Expression Data Using Real-Time Quantitative PCR and the 2^−ΔΔCT^ Method. Methods.

