# Supplementary figures and images for: Kyoh® Rocket Leaf Extract Regulates Proliferation and VEGF and FGF7 Expression in Human Dermal Follicle Papilla Cells
Source: Molecules. 2025 Mar 27;30(7):1489. doi: 10.3390/molecules30071489 (PMC11990418; doi:10.3390/molecules30071489)

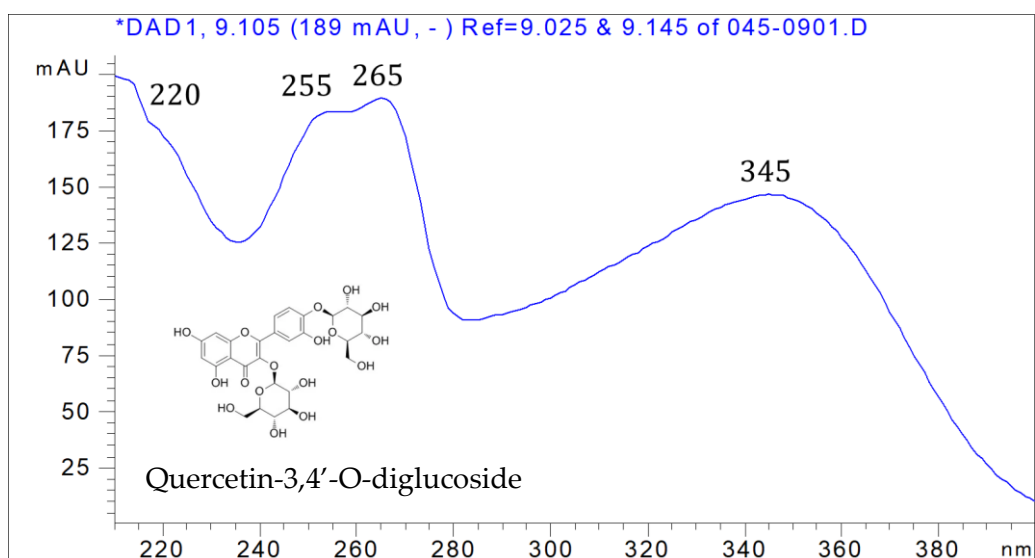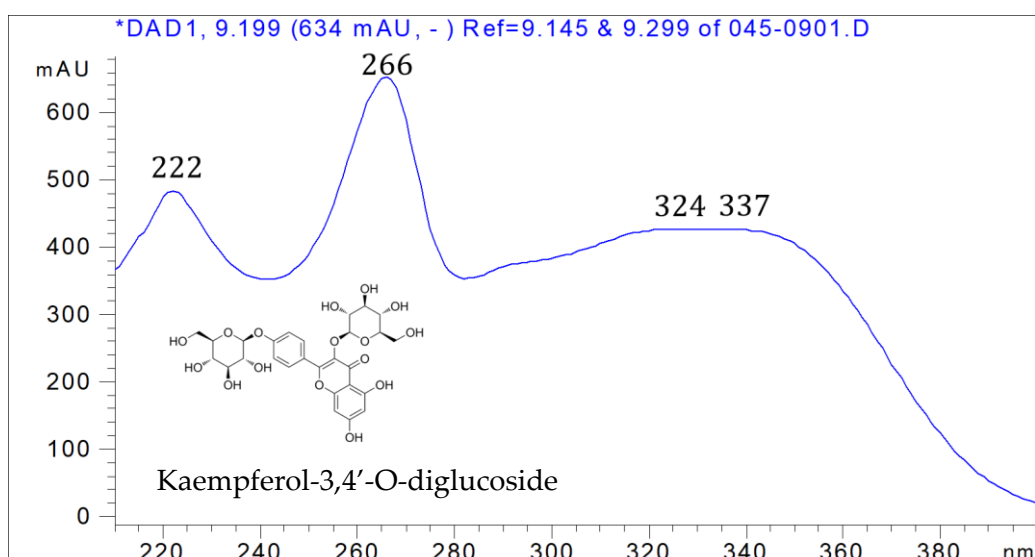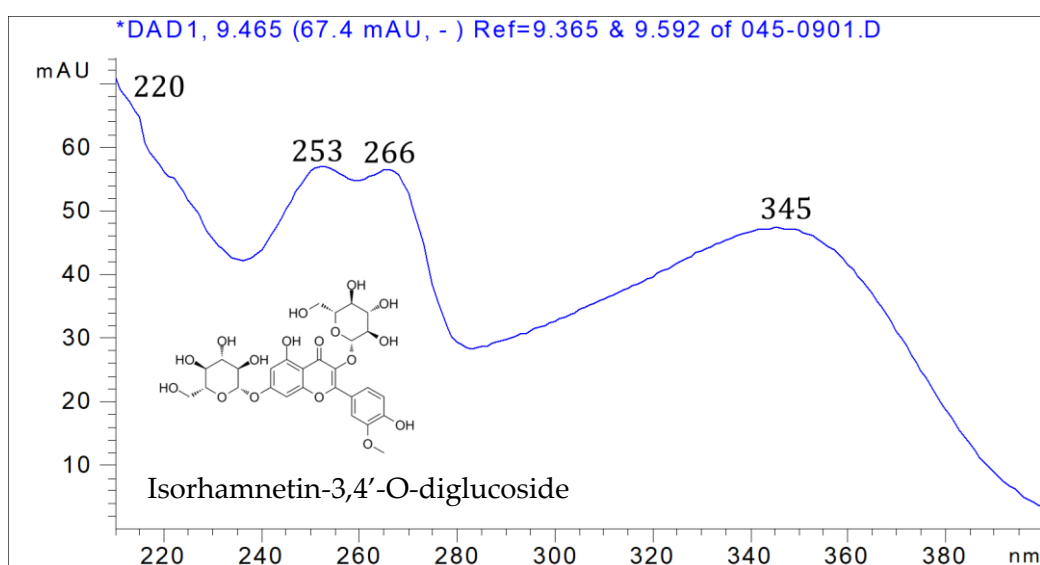

Figure S1. UV spectra of most abundant flavonols identified in Kyoh® extract.

Supplement: Supplementary file 1 [file molecules-30-01489-s001.zip › molecules-3486573-supplementary.pdf]
